# Brain networks underlying the processing of sound symbolism related to softness perception

**DOI:** 10.1038/s41598-021-86328-6

**Published:** 2021-04-01

**Authors:** Ryo Kitada, Jinhwan Kwon, Ryuichi Doizaki, Eri Nakagawa, Tsubasa Tanigawa, Hiroyuki Kajimoto, Norihiro Sadato, Maki Sakamoto

**Affiliations:** 1 Division of Psychology, School of Social Sciences, Nanyang Technological University, 48 Nanyang Avenue, Singapore, 639818 Singapore; 2 Faculty of Intercultural Studies, Kobe University, 1-2-1 TsuruKabuto, Nada-ku, Kobe, 657-8501 Japan; 3 Kyoto University of Education, Fukakusa-Fujimori-cho 1, Fushimi-ku, Kyoto, 612-8522 Japan; 4 Department of Informatics, Graduate School of Informatics and Engineering, The University of Electro-Communications, 1-5-1 Chofugaoka, Chofu, Tokyo, 182-8585 Japan; 5 National Institute for Physiological Sciences, Nishigonaka 38, Myodaiji-cho, Okazaki, 444-8585 Japan; 6 The Graduate University for Advanced Studies (SOKENDAI), Shonan Village, Hayama, Kanagawa 240-0193 Japan

**Keywords:** Sensory processing, Human behaviour

## Abstract

Unlike the assumption of modern linguistics, there is non-arbitrary association between sound and meaning in sound symbolic words. Neuroimaging studies have suggested the unique contribution of the superior temporal sulcus to the processing of sound symbolism. However, because these findings are limited to the mapping between sound symbolism and visually presented objects, the processing of sound symbolic information may also involve the sensory-modality dependent mechanisms. Here, we conducted a functional magnetic resonance imaging experiment to test whether the brain regions engaged in the tactile processing of object properties are also involved in mapping sound symbolic information with tactually perceived object properties. Thirty-two healthy subjects conducted a matching task in which they judged the congruency between softness perceived by touch and softness associated with sound symbolic words. Congruency effect was observed in the orbitofrontal cortex, inferior frontal gyrus, insula, medial superior frontal gyrus, cingulate gyrus, and cerebellum. This effect in the insula and medial superior frontal gyri was overlapped with softness-related activity that was separately measured in the same subjects in the tactile experiment. These results indicate that the insula and medial superior frontal gyrus play a role in processing sound symbolic information and relating it to the tactile softness information.

## Introduction

In modern linguistics, it is widely assumed that the acoustic features of a word are arbitrarily associated with its meaning^1,2^. This assumption is supported by the fact that the same concept is expressed by different sounds in different languages. However, an increasing number of studies have shown that a non-arbitrary association between sound and meaning can exist in some words called sound symbolic words^3–8^. For instance, a curvy round shape and spiky angular shape can be associated with the nonsense word “maluma” or “bouba” and with the nonsense word “takete” or “kiki”, respectively (the “bouba/kiki effect”)^8^. However, the neural mechanisms underlying this non-arbitrary mapping between sound and its semantic dimensions are not well understood.

Because sound symbolic words are associated with specific semantic dimensions, the processing of such words can involve the neural substrates for processing the corresponding semantic dimensions. For instance, if the sound symbolic words are associated with object shape and size, then the processing of these words can involve the neural substrates that associate the sound with the sensory processing of object shape and size. This idea is supported by findings which showed that congenitally blind individuals show weaker sound-shape associations^9–11^ and a study that found that prelingual auditory deprivation reduced the bouba–kiki effect, although they performed above chance level^12^. These findings raise the possibility that the processing of sound symbolic words associated with object properties involves sensory-modality dependent brain networks.

Previous neuroimaging studies have examined the brain networks that are involved in the processing of sound symbolic words^7,13–19^. Among them, more recent neuroimaging studies have examined brain activity when the subjects conducted matching tasks between sound symbolic words and visually presented objects such as: matching between Japanese mimetic words and body gestures^16^; matching between the size of visual stimuli and sound “bobo”/”pipi”^18^; and matching between sound “bouba”/”kiki” and the spikiness/roundness of visual stimulus^19^. Two of these studies showed that the matching between sound symbolic words and visually presented objects involves the region in and around the posterior part of the superior temporal sulcus (e.g., the middle temporal gyrus^18^), though the exact location of activation varied among the studies^16,18^. Kanero et al.^16^ proposed that this region is a part of the unique neural networks that process the sound symbolism. On the other hand, several studies also reported the activation of sensory-dependent networks in the processing of sound symbolic words. For instance, a study also reported that regions in the occipital cortex can be sensitive to incongruency between the visually presented object size and its sound^18^. Another study showed that the imagery of unpleasantness from pain-related mimetic words evoked activation of several brain regions including the anterior cingulate cortex, a part of the network of pain processing^14^. These findings partially support the hypothesis that the processing of sound symbolic words can involve sensory-dependent and sensory-independent networks in the brain.

The majority of the aforementioned neuroimaging studies have focused on the mapping between visually presented objects and sound symbolic words. Thus, it is unclear if the same neural substrates are involved in the matching between sound symbolic words and objects presented in other sensory modalities. In the present study, we focused our investigation on the neural correlates of association between sound symbolic words and object properties perceived by touch.

Previous neuroimaging studies have shown that specific sets of brain regions are engaged in tactile processing of object properties. Tangible object properties are organized into two major categories: macro-spatial and material properties^20^. The former category, which includes the perception of shape, orientation, and location, needs some form of a spatial reference system (spatial coding)^21^. By contrast, the latter category, which includes roughness, softness, and temperature, is expressed as intensity (intensity coding). It has been demonstrated that distinct but overlapping brain networks are involved in the processing of macro-spatial and material properties^22–24^. Specifically, activity in the parietal operculum (including the secondary somatosensory cortex, PO), insula, and occipital cortex is greater for texture perception than for perception of shape^23^ and of dot location on a cardboard^24^. The activation pattern in the parietal operculum and insula is related to the magnitude of perceived roughness^25,26^ and temperature^27^, though the ascending pathways for mechanical and thermal inputs differ. A recent functional magnetic resonance imaging (fMRI) study found that softness magnitude perceived by touch is represented in the network including the posterior insula, anterior insula, parietal operculum, and medial superior frontal gyrus^28^. Collectively, these studies indicate that the insula and PO are key nodes of the network for tactile perception of material properties^29,30^. Thus, if the sensory-dependent network plays a key role in the understanding sound symbolic information, the insula and PO can be also involved in the processing of sound symbolic words for material properties. Though a previous behavioral study showed that high pitched sounds are matched to haptically perceived angular shape and softness^10^, the underlying neural substrates have not been investigated. Thus, it remains unknown whether this network involving the insula is also associated with the mapping between tactile material information and sound symbolic words.

Another issue regarding the sound symbolic word is the familiarity with such words. If conventional sound symbolic words evoke brain regions that are related to sensory information, it can be based on the learned arbitrary rules or inherent non-arbitrary associations. Previous functional MRI studies used either conventional words^14,16^ or unfamiliar words^18,19^. However, to the best of our knowledge, the effect of familiarity of sound symbolic words has not been investigated. If a specific sound is associated with specific object properties, we can expect common brain networks involved in the processing of sound symbolic information, regardless of the familiarity.

In this study, we tested the hypothesis that the mapping between sound symbolic words and tactually perceived softness involves the insula/PO and medial superior frontal gyrus as the softness-dependent regions^28^ and the region in and around the posterior superior temporal sulcus (pSTS) as the sensory modality independent region^16,18^. To this end, we conducted a functional MRI study that involves the three tasks: matching task (main task), tactile task, and word softness-judgment task (word task).

**Figure 1:**
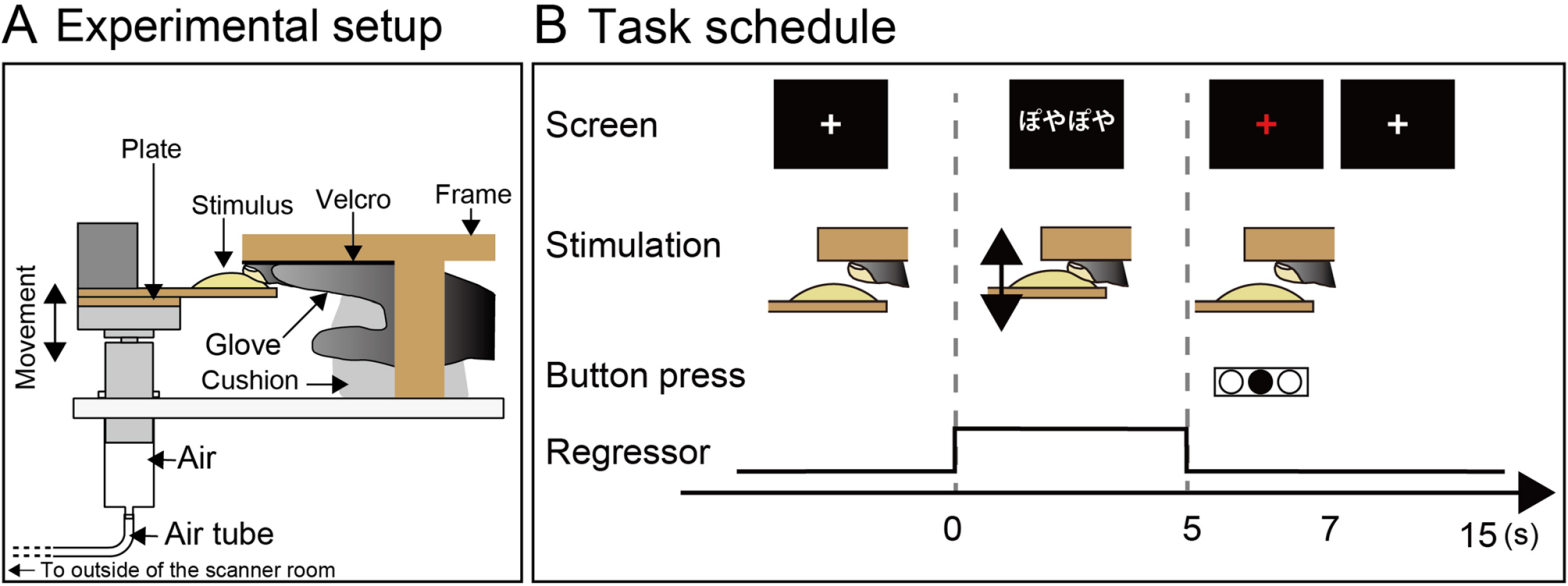
Experimental design. (**A**) Setup. We used an apparatus consisting of two cylinders that are connected via an air tube. A weight that was placed on the cylinder outside the scanner (not shown in the figure) pushes the cylinder inside the scanner via the air tube, which moved the stimulus onto the subject’s middle finger. The tactile stimuli had identical shapes and sizes, whereas their compliances, deformation per applied force, were varied. (**B**) Task schedule. In each trial, the fixation cross was replaced with one of the sound symbolic words, and the tactile stimulus was pushed onto the finger twice within 5 s. The subject was asked to judge the congruency of softness/hardness between tactile stimulus and sound symbolic words by pressing a button when the red fixation cross was presented. In the control condition in which randomized characters were presented and no tactile stimulus was given, the subject was asked not to respond.

The purpose of the matching task was to examine the neural correlates for the mapping between sound symbolic words and associated softness information. This design was adopted from previous studies on sound symbolic words^16,18,19^ and visuo-haptic association of material information^31^. Specifically, the task design includes two factors: the match between tactile and sound symbolic information and familiarity of sound symbolic words (Fig. 1). We then compared the conditions in which tactually perceived softness is matched to sound symbolic words (congruent conditions) with the conditions in which tactually perceived softness and sound symbolic words are mismatched (incongruent conditions). The assumption is that the interaction of the tactile information and sound symbolic information occurs to compare the two types of information in the hypothesized brain regions. We manipulated the familiarity of sound symbolic words by using genetic algorithms^32^.

Additionally, the two supplementary experiments (tactile and word judgment tasks) were conducted to examine signals when only tactile or sound symbolic information was given to the subjects. We assumed that activity of the hypothesized regions shows response even when only one type of the inputs was given.

## Results

Thirty-two healthy volunteers participated in the study. The matching task included five conditions: congruent pairs of tactile stimuli and familiar words, congruent pairs of tactile stimuli and unfamiliar words, incongruent pairs containing familiar words, incongruent pairs including unfamiliar words, and the low-level control condition. In the low-level control condition, only pseudowords were presented without tactile stimulation. The list of the sound symbolic words is available in the Supplementary material (Supplementary Table 1).

### Task performance

#### Matching task

Table 1 shows the congruency ratings and response times in the matching task. Two-way repeated-measures analysis of variance (ANOVA) (2 levels of matching × 2 levels of familiarity) for rating revealed a significant main effect of matching [F(1, 31) = 1629.3, *p* < 0.001] and a significant main effect of familiarity [F(1, 31) = 6.02, *p* = 0.02]. The same analysis also showed a significant interaction [F(1, 31) = 105.8, *p* < 0.001] with the effect of matching on ratings was greater for the familiar condition than the unfamiliar condition. Nevertheless, the post-hoc paired t-tests with Bonferroni correction confirmed that the rating was greater for congruency than for incongruency pairs, regardless of the familiarity (*p* values < 0.001).

**Table 1:** Behavioral results.

	Incongruent conditions		Congruent conditions	
	Unfamiliar	Familiar	Unfamiliar	Familiar
Congruency rating	1.45	1.20	3.27	3.65
SEM	0.04	0.03	0.05	0.04
Response time (s)	5.84	5.79	5.85	5.81
SEM	0.05	0.04	0.05	0.04

Response time is the time relative to the onset of each trial.

*SEM* standard error of the mean.

The same ANOVA for response time showed only a significant main effect of familiarity [F(1, 31) = 14.67, *p* = 0.001]. A significant main effect was observed neither for matching nor its interaction. Post-hoc paired t-tests with Bonferroni correction showed greater response time for pairs with unfamiliar words than for those with familiar words (*p* values < 0.05).

#### Word judgment task

Two-way repeated-measures ANOVA (2 levels of softness × 2 levels of familiarity) on rating showed significant main effects of softness [F(1, 30) = 2498.62, *p* < 0.001] and familiarity [F(1, 30) = 9.08, *p* = 0.005]. However the same ANOVA also showed a significant interaction between the two factors [F(1, 30) = 268.41, *p* < 0.001] with the difference of rating between “soft” and “hard” words in the familiar condition being greater than the same difference of rating in the unfamiliar condition. Paired t-tests with Bonferroni correction confirmed that softness ratings for words associated with softness were greater than those related to hardness, regardless of the familiarity (*p* values < 0.001) (Supplementary Table 2).

#### Tactile task

The behavioral ratings for the tactile task were shown in a previous study^18^. Specifically, we confirmed that the rating for the stimulus with higher compliance was greater than the stimulus with lower compliance in all pairs of stimuli.

### fMRI results

#### Matching task

##### Main effects of matching

**Figure 2:**
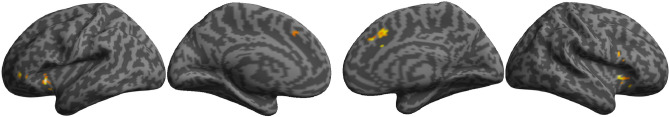
Main effects of matching (congruency effect). The activation revealed by the main effect of matching (congruency effect) in the group analysis was superimposed on a surface-rendered T1-weighted high-resolution MRI of an individual unrelated to the study. The opposite contrast revealed no significant activation. Note that no significant interaction with familiarity was observed. The statistical threshold for the spatial extent test was set at *p* < 0.05; family-wise error (FWE) corrected for multiple comparisons over the whole brain when the height (cluster-forming) threshold was set at *p* < 0.001 (uncorrected).

As hypothesized, the contrast of congruent conditions with incongruent conditions (congruency effect) revealed regions of significant activation in both the bilateral insula and medial superior frontal gyrus. Additionally, the same contrast revealed activation in the bilateral inferior frontal gyrus, left orbitofrontal cortex, bilateral anterior insula, right cingulate gyrus, and right cerebellum (Fig. 2, Supplementary Table 3). A significant effect was not observed in the posterior temporal regions (e.g., middle temporal gyrus). The opposite contrast revealed no significant activation.

##### Main effects of familiarity

**Figure 3:**
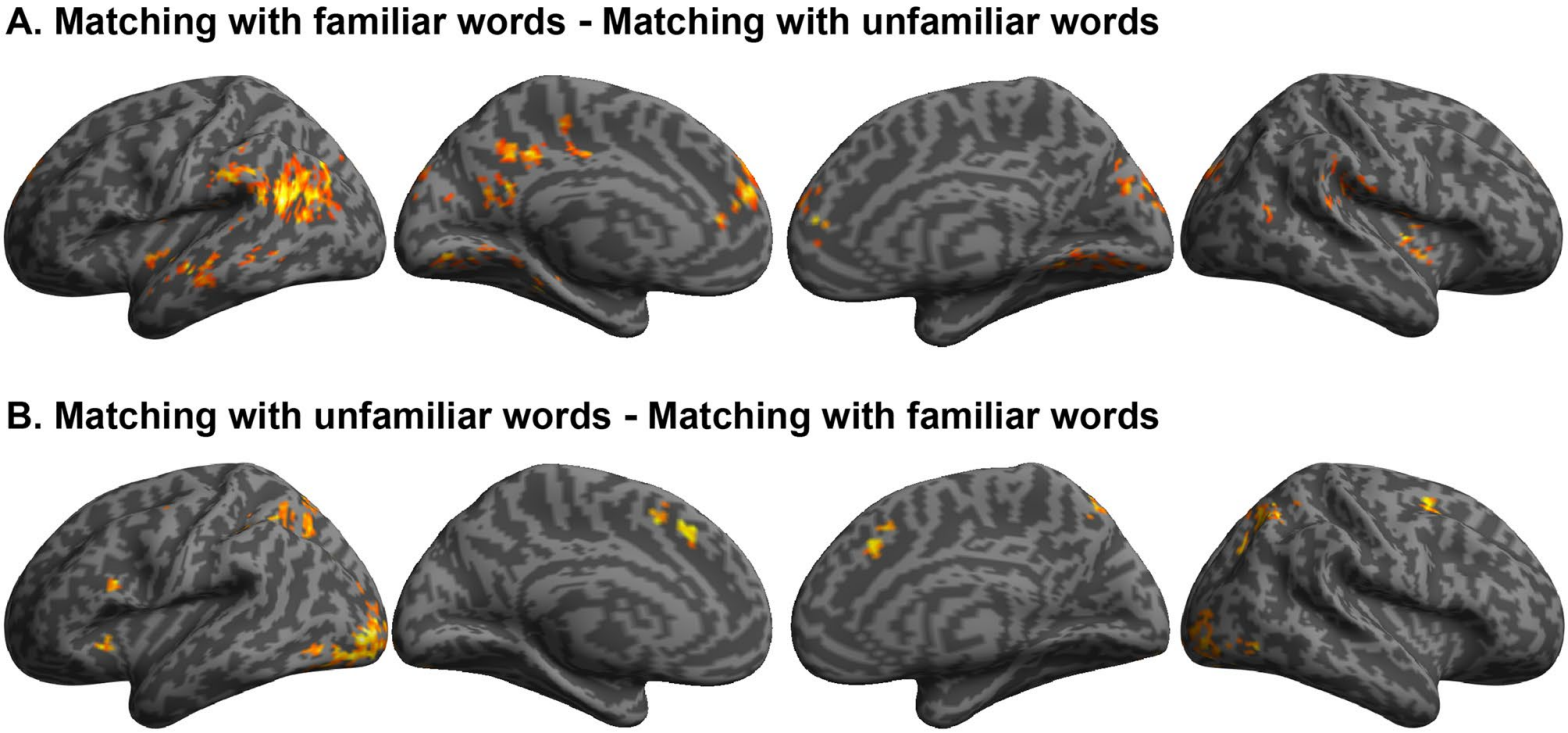
Main effects of familiarity (familiar conditions–unfamiliar conditions) in the matching task. The group-level activation revealed by the contrast of familiar sound symbolic words against unfamiliar words and by the opposite contrast was superimposed on a surface-rendered T1-weighted MRI of an individual unrelated to the study. No significant interaction with matching was observed. The statistical threshold for the spatial extent test was set at *p* < 0.05; family-wise error (FWE) corrected for multiple comparisons over the whole brain when the height (cluster-forming) threshold was set at *p* < 0.001 (uncorrected).

The contrast of conditions with familiar words against conditions with unfamiliar words revealed regions of significant activation bilaterally in the angular gyrus, cuneus, insula, lingual gyrus, middle temporal gyrus, precuneus, superior frontal gyrus, superior occipital gyrus, superior temporal gyrus, and supramarginal gyrus. Moreover, the same contrast showed activation in the left cingulate gyrus, left fusiform gyrus, left parahippocampal gyrus, left postcentral gyrus, left precentral gyrus, right middle occipital gyrus, and right parietal operculum (Fig. 3A, Supplementary Table 4).

The opposite contrast revealed regions of significant activation in the bilateral angular gyrus, bilateral inferior occipital gyrus, bilateral middle frontal gyrus, bilateral middle occipital gyrus, bilateral superior frontal gyrus, bilateral superior occipital gyrus, bilateral superior parietal lobule, left inferior frontal gyrus, left insula, left supramarginal gyrus, right precuneus, and bilateral cerebellum (Fig. 3B, Supplementary Table 4).

##### Interaction between matching and familiarity effects

No significant activation was observed.

#### Tactile task

**Figure 4:**
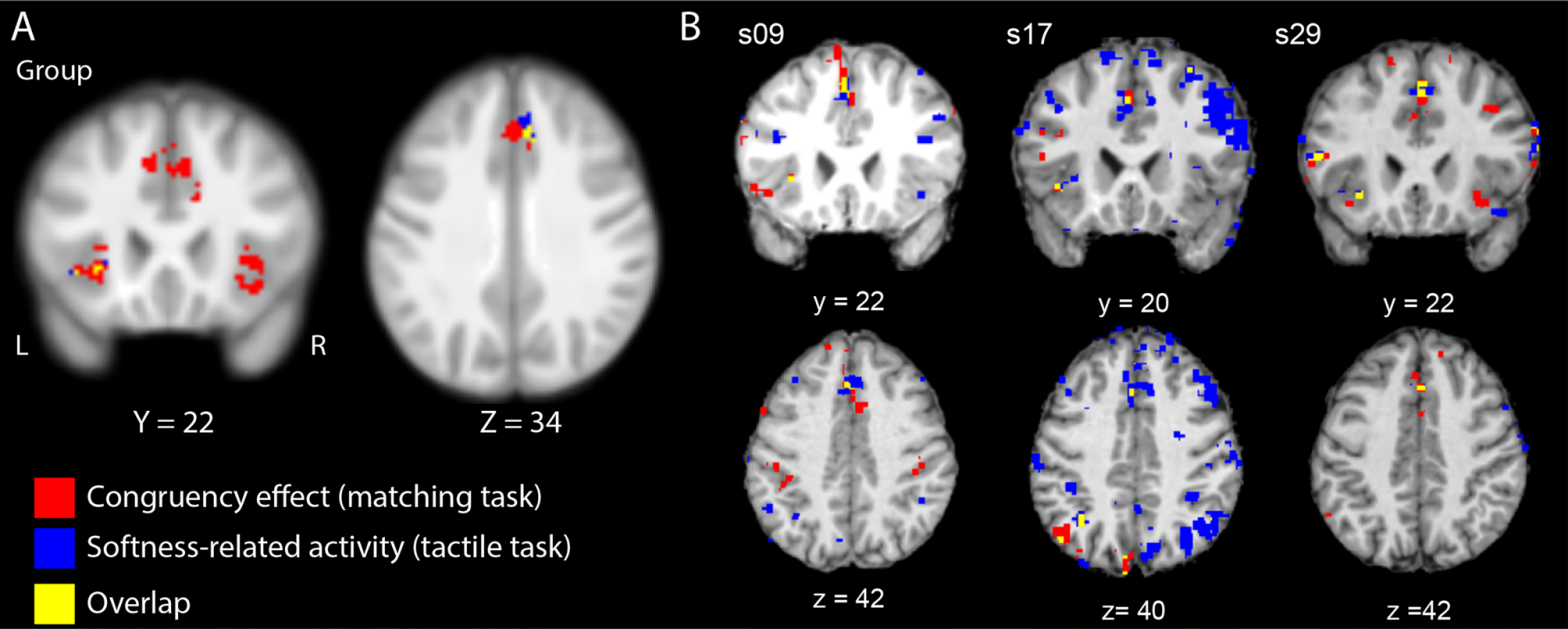
Overlap between the congruency effect in the matching task and the softness-related activation in the tactile task. (**A**) Overlap of activation at the group-level between the congruency effect in the matching task (Fig. 2) and the softness-related activation revealed by the parametric-modulation analysis of the tactile task. The activation was presented on coronal and horizontal sections of the MRI averaged over the subjects. The statistical threshold for the spatial extent test was set at *p* < 0.05; family-wise error (FWE) corrected for multiple comparisons over the whole brain when the height (cluster-forming) threshold was set at *p* < 0.001 (uncorrected). (**B**) Representative individual results of overlapped activation. The height threshold was set at *p* < 0.001 (uncorrected) without a cluster threshold.

The contrast for parametric-modulation revealed activation in the left insula, parietal operculum, and medial superior frontal gyrus. As shown in Fig. 4A, the congruency effect in the matching task was overlapped with the softness-related activation in the tactile task in the anterior insula and medial superior frontal gyrus. The conjunction analysis (with conjunction null) using an inclusive masking procedure^33,34^ confirmed significant activation in the left anterior insula and medial superior frontal gyrus (FWE corrected *p* values < 0.05). Figure 4B shows the data of three representative subjects.

#### Word judgment task

The contrast of judgment of sound symbolic words against baseline revealed multiple regions of significant activation over the whole brain, including the bilateral insula, medial superior frontal gyrus, and pSTS (Supplemental Fig. 1A). The contrast of the interaction between softness impression and familiarity [(Familiar_Soft–Familiar_Hard) − (Unfamiliar_Soft–Unfamiliar_Hard)] revealed no significant activation in the hypothesized regions; instead it revealed regions of significant activation in the bilateral lingual gyrus, bilateral middle occipital gyrus, bilateral cuneus, and left inferior occipital gyrus. Given the presence of significant interaction, we evaluated the effect of softness in each level of familiarity. As compared to the hard unfamiliar word condition, the soft unfamiliar word condition revealed greater activation in the bilateral lingual gyrus, bilateral middle occipital gyrus, bilateral cuneus, and left inferior occipital gyrus (Supplementary Fig. 1B–E, Supplementary Table 5). By contrast, no brain region showed greater activation in the soft familiar word condition than in the hard familiar word condition. Collectively, no significant effect of softness-hardness impressions in the hypothesized regions was observed in the whole brain analysis.

#### VOI analysis

**Figure 5:**
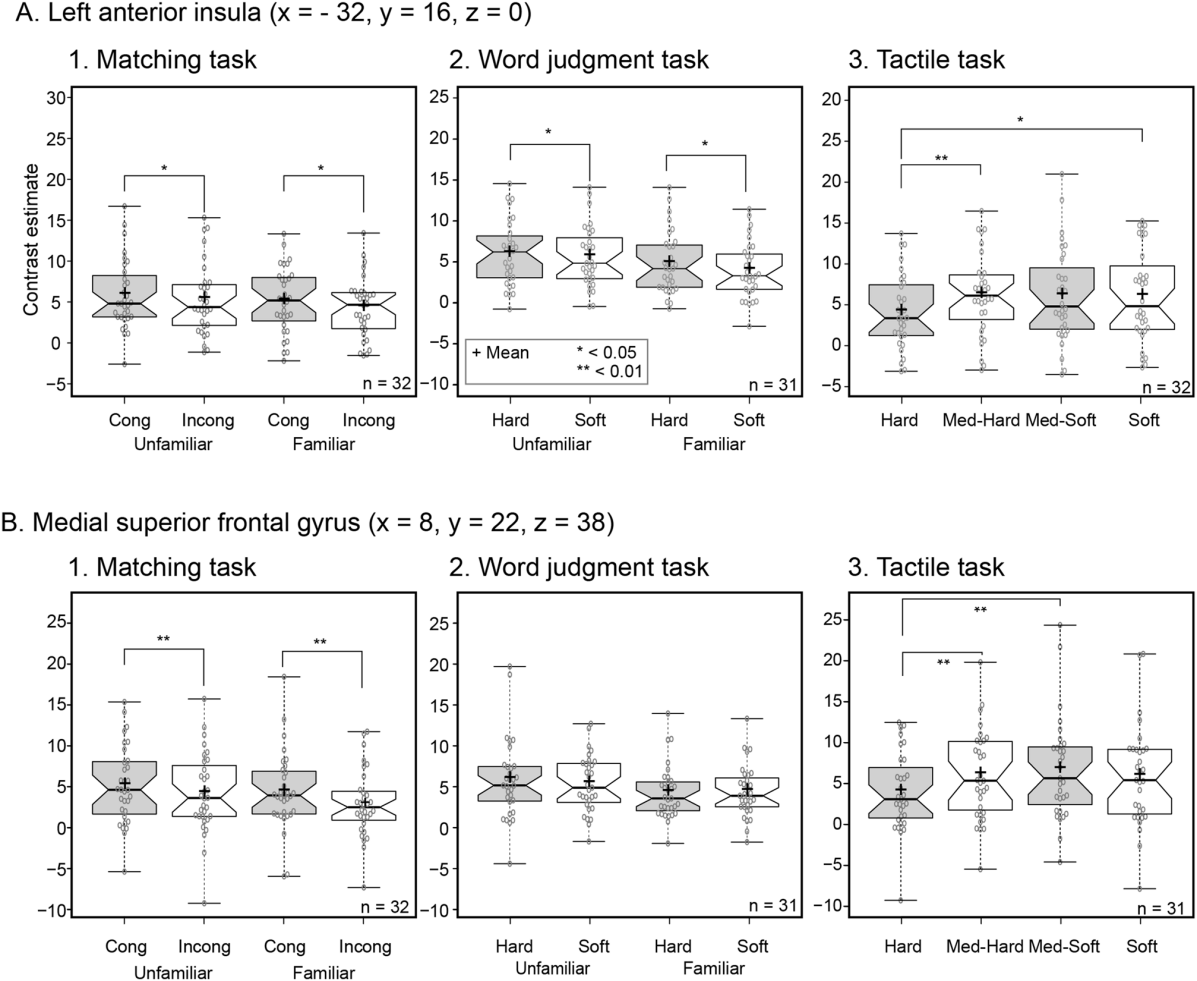
Volume-of-interest analysis. Activity (parameter or contrast estimates) extracted from the volumes of interest (VOIs) that were defined with the independent data set (the main experiment in Kitada et al.^28^). Data are presented as boxplots, where the box indicates the interquartile range [IQR, from 25th percentile (Q1) to 75th percentile (Q3)]. The thick line and black cross in the box indicate the median and mean, respectively. Whiskers indicate the maximum and minimum value. Circles and notches of the boxes indicate data points and 95% confidence levels, respectively. Asterisks and symbols indicate the result of ANOVA for the matching and word judgment tasks, and the result of post-hoc pairwise comparisons (with Bonferroni correction) for the tactile task. Note that one subject’s data was excluded from the analysis of the word judgment task due to technical issues. The figure was made with BoxPlotR (http://shiny.chemgrid.org/boxplotr/).

Next, we conducted volume-of-interests (VOI) analysis to examine the activation patterns in the hypothesized regions (insula and the medial parts of the superior frontal gyrus) across the three tasks (Fig. 5). To minimize the selection bias that using the same dataset for selection and selective analysis cause (“double-dipping” problem)^35^, we used two independent data sets. One data set originated from our previous study^28^ and was used to localize peak coordinates in these hypothesized regions. Then, we examined activity in these coordinates in the other data set that included the tasks in the present study.

##### Matching task

Two-way repeated-measures ANOVA (2 levels of matching × 2 levels of familiarity) on the contrast estimates (relative to the control) revealed significant main effects of matching, with the congruent condition showing greater contrast estimates than the incongruent condition in both regions [F(1,31) = 6.2, *p* = 0.019 for the left insula; F(1, 31) = 25.2, *p* < 0.001 for the superior frontal gyrus]. The same analysis showed significant main effects of familiarity, with unfamiliar conditions showing greater activation than familiar conditions in both regions [F(1,31) = 5.7, *p* = 0.023 for the insula; F(1, 30) = 11.8, *p* = 0.002 for the superior frontal gyrus]. None of the ROIs showed a significant interaction effect (*p* values > 0.4).

##### Word judgment task

Two-way repeated-measures ANOVA (2 levels of softness × 2 levels of familiarity) on parameter estimates revealed significant main effect of softness with hard words producing greater activity than soft words in the insula [F(1, 30) = 6.3, *p* = 0.018]; main effects of familiarity with unfamiliar words producing greater activity in the all ROIs [F(1, 30) = 23.7, *p* < 0.001 for the left insula; F(1, 30) = 14.5, *p* = 0.001 for the superior frontal gyrus]. None of the regions showed significant interaction (*p* values > 0.1).

##### Tactile task

Two-way repeated-measures ANOVA (4 levels of softness) on the contrast estimates (relative to the control) revealed a significant main effect in all regions [F(3, 93) = 4.7, *p* = 0.004 for the insula; F(3, 93) = 7.1, *p* < 0.001 for the superior frontal gyrus]. Pairwise comparisons with Bonferroni correction showed that the contrast estimates for the hardest stimulus were lower than those for the second-hardest (Med-Hard) stimulus in both regions (*p* values < 0.01), lower than those for the softest stimulus in the left insula (*p* = 0.033), and lower than those for the second-softest stimulus (Med-Soft) in the superior frontal gyrus (*p* = 0.007). These results confirm that the insula and superior frontal gyrus are affected by the magnitude of tactually perceived softness.

#### Multi-voxel pattern analysis

**Figure 6:**
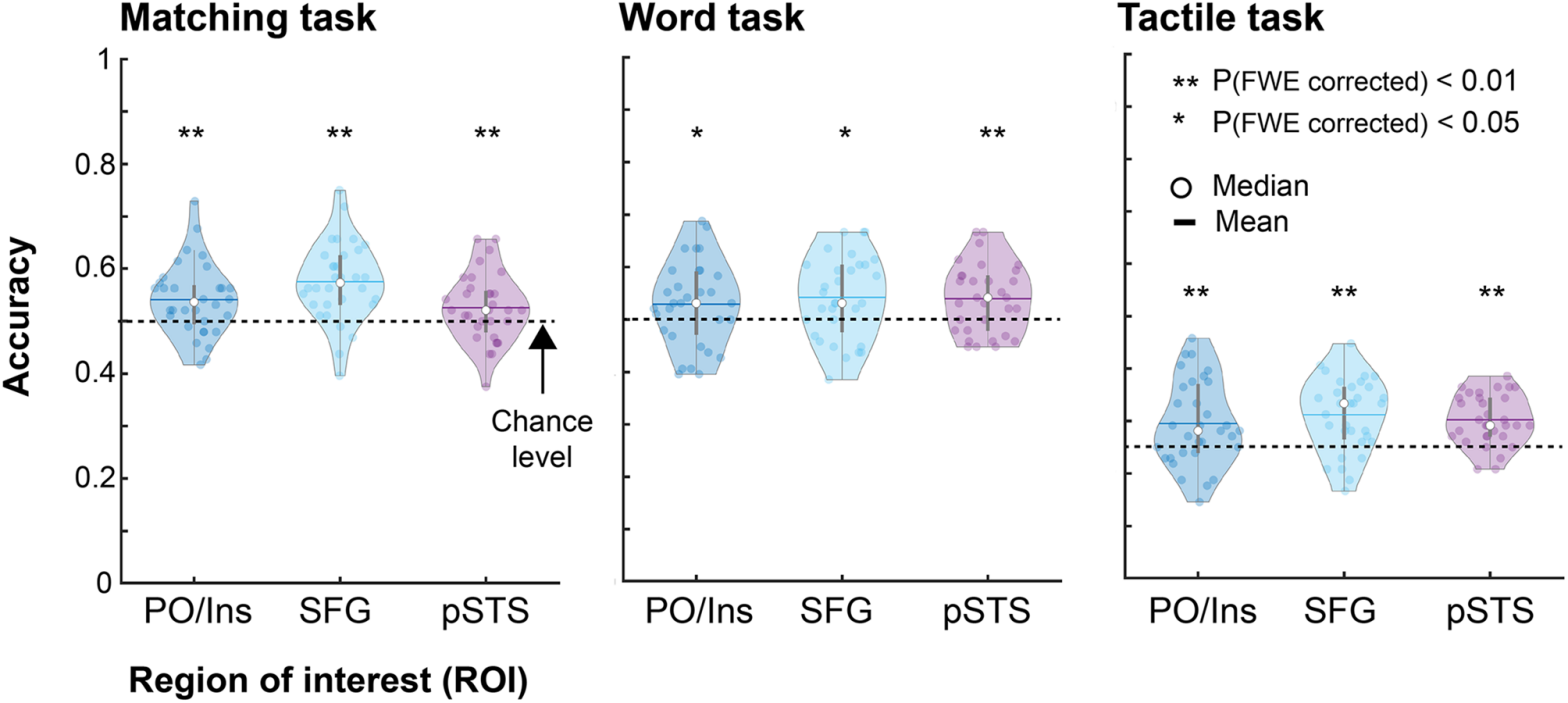
Multi-voxel pattern analysis on object compliance. Distributions of performance accuracy of MVPA. Each dot indicates the performance for each subject. Asterisks indicate the result of permutation tests (with Bonferroni correction). PO/Ins, parietal operculum/insula; SFG, medial superior frontal gyrus; pSTS, region in and around the superior temporal sulcus; FWE, family-wise error. Note that dot lines indicate the theoretical chance level: 50% for the matching and word judgment tasks and 25% for the tactile task.

The univariate anlaysis showed the matching effect in the insula nad medial superior frontal gyrus, whereas no such effect was found in the pSTS. To further examine whether the pSTS contains information about sound symbolic information and its matching effect with tactile information, we conducted a multi-voxel pattern analysis (MVPA) (Fig. 6). Based on our previous studies^16,18,28^ the insula/PO, medial part of superior frontal gyrus, and pSTS were chosen as ROIs. All ROIs in all tasks showed significantly greater accuracy than chance level (*p* values < 0.05 Bonferroni corrected).

## Discussion

In this study, we examined the brain networks engaged in the processing of sound symbolic information that are associated with object’s softness and hardness. While a distributed set of brain regions was affected by the congruency between perceived softness and sound symbolic words, the congruency effect was observed in or close to the region that showed the graded response to perceived softness in the anterior insula and medial superior frontal gyrus. This effect was similarly observed regardless of the familiarity (i.e., no interaction effect between familiarity and matching). The MVPA showed that the PO/insula contained information on congruency, the magnitude of softness perceived by touch, and the magnitude of softness associated with sound symbolic words.

This study used different words between conditions. The matching task involved the same sets of words and tactile stimuli between the congruency and incongruency conditions. The subtraction of the incongruency effect from the congruency effect should cancel out the difference of these inputs. Thus, it is unlikely that the observed congruency effect is explained merely by the difference in stimuli. We used two independent data sets for the VOI and ROI analysis: one data set was used to localize the brain regions for tactile softness perception in VOI and ROI analysis from our previous study^28^ and the other data set which consist of the three tasks (matching task, tactile task, and word judgement task) were used to evaluate activity in these identified regions. This procedure follows the policy of selective analysis^35^ as used in previous studies^18,36,37^. Thus, we have minimized selection bias in our procedure.

To the best of our knowledge, this is the first neuroimaging study that depicted the interaction of brain activity between sound symbolic words and material properties perceived by touch. More specifically, previous neuroimaging studies highlighted the involvement of the pSTS in the matching between visually presented body action and mimetic words^16^ and between the size of visual stimuli and auditorily presented onomatopoeia^18^. In contrast, the present study revealed that the insula and medial superior frontal gyrus are associated with the congruency effect between tactile softness perception and sound symbolic words. This network contained information on the perceived magnitude of softness, as well as the congruency effect. This result indicates that the brain network that processes information of perceived softness is also engaged in processing the associated sound symbolic words. This finding is consistent with the idea that the processing of sound symbolic information involves the sensory-modality dependent regions. For instance, the onomatopoeia of pain can evoke brain activation in the anterior cingulate cortex, which is considered a part of the pain processing network^14^. The comparison of visual sizes of stimuli with sound symbolic words activated the occipital lobe as well as the pSTS^18^.

Within the regions showing the congruency effect, the left insula contained some information about sound symbolic words, as evidenced by the VOI and MVPA analyses on the word judgment task. Thus, it is possible that the insula receives the information about softness perceived by touch and softness associated with sound symbolic words, causing the interaction between the two types of information. This speculation is fit to the concept of multisensory integration that occurs in a region with signals of each sensory modality^38,39^.

We also found the congruency effect in other prefrontal regions, consistent with the previous finding of an intermodal interaction effect in the prefrontal cortex^6,7^. In our stimuli, the sounds /k/ and /g/ were associated with words related to hard referents (e.g., kada-kada and gai-gai), whereas the sounds /p/ and /f/ were associated with words related to soft objects (e.g., funo-funo and yapu-yapu). To confirm the associated tactile softness, some subjects reported that they covertly produced the sounds of the presented words. Thus, activation in these regions may be partly associated with such heuristics. This speculation is consistent with the perspective that the anterior insula and inferior frontal gyrus are associated with covert articulation^40^, as well as with previous findings that this region is also sensitive to matching between picture and words that do not contain sound symbolism^41–43^. The congruency between the impression of softness from covertly-produced sounds and tactile information may become more salient, leading to activation in the insula and the medial areas in the superior frontal gyrus. This idea is in accord with the view that the anterior insula and medial prefrontal cortex form the network for salient stimuli (saliency network) ^44,45^.

The MVPA also showed that the pSTS contains information about congruency. This indicates that the posterior temporal region is also a part of the network in matching between tactile stimuli and sound symbolic information. However, contrary to the findings in previous studies^16,18^, this region showed a nonsignificant effect in the mass-univariate analysis. One interpretation for this weak effect is the difference in the stimuli. Again, the comparison of sensory stimulus with the sound symbolic information should occur in the regions that receive inputs from each related network. For instance, Kanero et al.^16^ used body actions as the referents of mimetic words. The pSTS is a key node of the network of action understanding^46–48^ as well as language processing^48,49^; hence, a suitable area for the matching effect. Likewise, other studies have shown an interaction effect in a distributed set of the occipital and temporal cortex, which is ideal for auditory-visual integration^18,19^. By contrast, our study involved tactile softness perception, which involves a network of frontal regions rather than the posterior temporal region^28^. Thus, it is possible that the pSTS plays a minor role in the matching between tactile stimuli and sound symbolic words, although it can be a part of the engaged network. Thus, further research is necessary to examine to what extent the pSTS plays a modality-independent role in processing sound symbolic information.

Familiar and unfamiliar sound symbolic words caused different patterns of brain activation in the matching between tactile and symbolic word information. Thus, our results indicate that, although mapping between tactile and sound symbolic words occurs in the same frontal regions, the familiarity affects the activation of cortical networks. The familiar sound symbolic words evoked activation in the brain regions including the infero-medial prefrontal cortex, angular gyrus, and posterior cingulate gyrus. This network for the recognition of familiar sound symbolic words is highly similar to that in the results of a meta-analysis on familiarity^50^. On the other hand, unfamiliar sound symbolic words showed stronger activation in a different set of brain regions. Our behavioral results showed that the effect of matching on ratings was greater for the familiar condition than the unfamiliar condition, and the response time for unfamiliar words was greater than that for familiar words. These findings indicates that it requires greater task demand to interpret unfamiliar words than familiar words. Thus, it is possible that greater activation in the unfamiliar condition may be due to the increased task demand to process novel information.

Finally, it is worth noting two interpretational issues. First, the word judgment task showed a higher signal for hardness than for softness in the left anterior insula, whereas activity in the tactile task was positively graded response to softness. Even though this region contains information about softness associated with the sound symbolic word, the similarities and differences between tactile and sound symbolic representation of softness in this region remain unclear. Thus, further research is necessary to address this point. Second, it is not clear to what extent our result can be generalized to tactile tasks, since we used a limited set of sound symbolic words and a single tactile stimuli. This is because fMRI studies with stringent control of tactile stimuli are technically challenging and it is difficult to use multiple object properties in one experimental setup (e.g., both roughness and softness). Future studies should examine to what extent the matching effect is generalized by using other sound symbolic words and tactile stimuli with perceptual dimensions other than softness.

In conclusion, the insula and the medial parts of the superior frontal gyrus, which were associated with softness magnitudes perceived by touch, showed a congruency effect between softness perceived by touch and softness associated with sound symbolic words. This result indicates that these regions constitute nodes of the network for mapping sound symbolic information onto tactile material information. In contrast to the previous findings on the neural correlates for sound symbolic words, our finding highlights the role of the nodes in the prefrontal cortex for the network of the matching between sound symbolic information and tactile material information.

## Methods

### Subjects

Thirty-two Japanese individuals (17 men, 15 women) aged 18–35 years (mean ± standard deviation = 23.3 ± 4.9 years) participated in the study. All subjects were right-handed, as assessed using the Edinburgh Handiness Inventory^51^. None of the subjects reported a prior loss of tactile sensation or a history of major medical or neurological illnesses, such as epilepsy, significant head trauma, or a lifetime history of alcohol dependence.

The study was conducted in accordance with the Declaration of Helsinki. All subjects provided written informed consent before participation. The study protocol was approved by the ethics committees at the National Institute for Physiological Sciences. All procedures were performed in accordance with the approved guidelines.

### Experimental design and statistical analysis

The subjects performed three tasks: the main task, touch-word matching task (matching task), tactile softness-judgment task (tactile task), and word softness-judgment task (word judgment task). We adopted within-subject experimental designs in all tasks. All behavioral data were analyzed using SPSS software (version 23; IBM Corporation, Armonk, NY). Bonferroni correction was applied to control for multiple comparisons. All fMRI data were analyzed using the Statistical Parametric Mapping 12 (SPM12) software package^52^ (RRID: SCR_007037) in MATLAB (MathWorks, Natick, MA, USA). In the mass-univariate analysis, the statistical threshold for the spatial extent test on the clusters was set at *p* < 0.05, family-wise error (FWE) corrected for multiple comparisons over the whole brain. The height (cluster-forming) threshold was set at *p* < 0.001 (uncorrected). This threshold is sufficiently high to use the random-field theory to control FWE rate^53^. We used CoSMoMVPA toolbox to perform multi-variate voxel pattern analysis^54^ (RRID: SCR_014519).

### Stimulus presentation

#### Words

We prepared 60 words. Twelve sound symbolic words that refer to softness (Familiar_Soft) and 12 that refer to hardness of objects (Familiar_Hard) were selected from a dictionary of Japanese mimetic words^55^. For instance, “fuka-fuka” and “kachi-kachi” were included in the stimuli. In addition, we prepared 24 unfamiliar sound symbolic words, 12 for softness (Unfamiliar_Soft) and 12 for hardness (Unfamiliar_Hard) using genetic algorithms^32^. These new words include “yapu-yapu” for softness and “guko-guko” for hardness (Supplementary Table 2). The number of Japanese characters ranged from 4 to 6 in each category and the number of morae was limited to 2–3. Initially, 300 sound symbolic words were generated by the algorithm and then evaluated by 12 subjects (7 male and 5 female) who did not participate in this study. We selected 24 based on the result of this pilot experiment. Finally, we made 12 pseudowords by assigning 4 Japanese characters in a pseudo-randomized order (Random, e.g., “fugusau”).

#### Tactile stimuli presentation

We used 5 spherical segments that were made of urethane elastomer covered by a plastic membrane and 1 spherical segment made of fiberglass-reinforced plastic (Bioskin; Beaulax Co., Ltd., Tokyo, Japan), all of which had the same size (5-cm diameter base × 1.3-cm height). We selected four stimuli for the matching task based on their compliance^28^; the compliances for these stimuli were 10.26, 5.76, 0.79, and 0.20 mm/N. The former two stimuli were considered as soft, and the latter as hard stimuli. To examine the activity associated with moderately soft stimuli (i.e., between 1 and 5 mm/N), we used a different set of four stimuli for the tactile task (10.26, 4.27, 1.13, and 0.20 mm/N).

We used the same MRI-compatible stimulus presentation device used in our previous study (Fig. 1A)^28^. This device had a wooden frame supporting the subject’s right hand and pneumatic cylinders. The subjects wore a glove that had a hole at the tip of the right middle finger. The backside of the glove was fastened to a Velcro strip that was attached to the ceiling of the wooden frame. At the onset of each trial, the device pushed the stimuli upward onto the right middle finger. The duration of each stimulation was approximately 2.2 s, repeated twice within 5 s. One experimenter stood beside the scanner and wore MRI-compatible headphones (Kiyohara Optics, Tokyo, Japan).

#### Matching stimuli

We paired each sound symbolic word with both soft and hard stimuli, creating congruent and incongruent pairs. Each of the four tactile stimuli was paired with 6 words in each of the four categories (Unfamiliar_Hard, Unfamiliar_Soft, Familiar_Hard, and Familiar_Soft; 6 words × 4 categories = 24 words for each tactile stimuli). Consequently, we prepared 24 congruent pairs with familiar words, 24 congruent pairs with unfamiliar words, 24 incongruent pairs with familiar words, and 24 incongruent pairs with unfamiliar words. Each pair was presented only once in the experiment.

### Setup

The setup was identical to our previous study^28^. Subjects were placed in the supine position and were instructed to remain relaxed during the scanning. Their heads were fixed using foam pads and tape to minimize movement. The subjects were asked to extend their arms, placing their right hand in the wooden frame. They held a response box in their left hand. Visual stimuli were presented to the subjects using the Presentation software (Neurobehavioral Systems, Inc., Albany, CA, USA) implemented on a personal computer (dc7900; Hewlett-Packard, Ltd., Palo Alto, CA, USA). A liquid crystal display projector (CP-SX12000; Hitachi, Ltd., Chiyoda, Tokyo, Japan) located outside and behind the scanner projected the stimuli through a waveguide to a translucent screen, which the subjects viewed via a mirror placed on the MRI scanner. The same software was used to present auditory cues for the next stimulus to be presented and the timing of replacing the stimulus with the next one. The auditory cues were only presented to the experimenter via headphones.

### Data acquisition

We used a 3-T whole-body MRI scanner (Verio; Siemens, Erlangen, Germany) with a 32-element phased-array head coil. We employed a multiband echo-planar imaging (EPI) sequence that collected multiple slices simultaneously, reducing the repetition time (TR) per volume^56^. The same parameters as our previous study^28^ was used to cover the whole brain: gradient-echo EPI, TR = 1000 ms, multiband factor = 6, echo time (TE) = 35 ms, flip angle = 65°, 60 axial slices of 2-mm thickness with a 25% slice gap, field-of-view = 192 × 192 mm^2^, and in-plane resolution = 2.0 × 2.0 mm^2^. T1-weighted high-resolution anatomical images were acquired for each subject using magnetization-prepared rapid acquisition gradient-echo (MP-RAGE) sequences (TR = 1800 ms, TE = 1.98 ms, flip angle = 9°, and voxel size = 1 × 1 × 1 mm^3^).

### Matching task

The matching task included two factors: degree of match and familiarity of the sound symbolic word. Each subject completed 6 runs of the task, which included each of the five conditions: congruent pairs of tactile stimuli and familiar words, congruent pairs of tactile stimuli and unfamiliar words, incongruent pairs containing familiar words, incongruent pairs including unfamiliar words, and the control condition. In the low-level control condition, only pseudowords (Random) were presented without tactile stimulation.

A single run consisted of twenty 15-s trials (300 s in total) preceded by 20-s rest and followed by 10-s rest (300 + 20 + 10 = 330 s in total, 330 volumes). Each condition was repeated 4 times in a single run (4 repetitions × 6 runs = 24 trials for each condition). One of the four tactile stimuli was presented once for each condition in each run. The order of the five conditions in each run was pseudo-randomized. In each trial, a white cross was replaced with words for 5 s (Fig. 1B). During this period, the subject’s middle finger was stimulated in all conditions except for the control condition. After 5 s, the red cross was presented for 2 s, during which the subject pressed one of the four buttons with their left hand to indicate the degree of match (congruency) between the words and the tactile stimuli. The response was calculated as the degree of match from 1 (lowest congruency) to 4 (highest congruency). The order of the button presses was counterbalanced across the subjects. During the rest of the trial, the subject was asked to stay still and the experimenter replaced the stimuli.

#### Data processing and analysis

The first 15 volumes of each fMRI run were discarded to allow the MR signal to reach a state of equilibrium. The remaining volumes were used for the subsequent analyses. To correct for head motion, functional images from each run were realigned to the first image and again realigned to the mean image after the first realignment. The T1-weighted anatomical image was co-registered to the mean of all realigned images. Prior to co-registration, the T1-weighted anatomical image was skull-stripped to prevent non-brain tissue from affecting the alignment between the EPI and T1-weighted images. Each co-registered T1-weighted anatomical image was normalized to the Montreal Neurological Institute (MNI) space using the DARTEL procedure^57^. More specifically, each anatomical image was segmented into tissue class images using a unified segmentation approach. Gray and white matter images were registered and normalized to space using the preexisting template that is based on the data from 512 Japanese individuals scanned at the National Institute for Physiological Sciences, Japan. The parameters from DARTEL registration and normalization were then applied to each functional and T1-weighted anatomical image. The normalized functional images were filtered using a Gaussian kernel of 4-mm full-width at half-maximum (FWHM) in the *x*, *y*, and *z* axes. We then conducted mass-univariate analyses as explained below.

#### Mass-univariate analysis

A general linear model was fitted to the fMRI data for each subject. The blood-oxygen-level dependent (BOLD) signal for the period of stimulus presentation was modeled using boxcar functions convolved with the canonical hemodynamic response function. Each run in the design matrix included 4 task-related regressors for each task condition (2 levels of match × 2 levels of familiarity) and 1 control condition. The time series for each subject was high-pass-filtered at 1/128 Hz. As the traditional AR(1)+ white noise model can fail to whiten the data with short TR, temporal autocorrelations were modeled and estimated from the pooled active voxels by the FAST model and were used to whiten the data^58,59^. Motion-related artifacts were minimized by incorporating the 6 parameters (3 displacements and 3 rotations) from the rigid-body realignment stage into each model. The contrast estimates for the main effects of each factor and their interactions were evaluated using linear contrasts.

Contrast images from the individual analyses were used for the group analysis, with between-subject variance modeled as a random factor. The contrast images obtained from the individual analyses represent the normalized task-related increment of the MR signal of each subject. We performed one-sample t-tests on the contrast estimates obtained from the individual analyses. The resulting set of voxel values for each contrast constituted the SPM{t}. The search volume was the whole brain. Brain regions were anatomically defined and labeled in accordance with probabilistic atlases^60^ and an anatomical MR image averaged over all subjects.

#### VOI analysis

We conducted a VOI analysis to confirm the activation patterns in the brain areas that showed congruency effect in the main experiment and softness-related activity in the supplementary tasks (described below). We selected the coordinates by analyzing independent data on tactile softness perception^28^. Briefly, the experiment of this independent data was identical to the tactile task in the present experiment except that it adopted 2 within-subject factor design with 3 levels of softness and 2 levels of forces. To identify the coordinates that showed softness-related activation regardless of the force levels, we employed a full factorial design that includes all the task-related conditions. We used standardized scores of softnes ratings as contrast weights for each force condition. We extracted parameter estimates from peak coordinates found in this analysis.

#### Multi-voxel pattern analysis

We examined whether the predefined regions of interest (ROIs) contain information on matching between words and touched objects. We constructed new design matrices to obtain t-values for each trial. For each subject, 6 design matrices were produced with each modeling trials in each run. Each regressor in a design matrix modeled BOLD signal during the presentation of stimuli in each trial. Thus, each design matrix contained 20 task-related regressors (4 repetitions × 4 matching conditions + 4 repetitions per run for the control condition) as well as the 6 motion-related parameters. We generated a map of voxel-wise t-values (SPM{t} map) for each trial of each subject by evaluating the linear contrast of the regressor of each trial against implicit baseline.

We performed classification analyses on the voxel-wise t-values of each subject^61^. A linear support vector machine (MATLAB’s SVM) was trained on data obtained from 5 runs to predict matching (congruent or incongruent) in the remaining run. Accuracy was recorded for the attempted classification of the data. This process was repeated 6 times, using a different run as the test data (leave-one-run-out cross-validation). These cross-validated analyses were performed separately for each ROI. Because the conventional one-sample t-test against the theoretical chance level may not provide a valid population inference^62,63^, we used random permutation tests in each region at the single-subject level, and then combined the results at the group-level with a bootstrap method^63^. More specifically, we randomly shuffled categories for each t map and then conducted the aforementioned procedure 1000 times for each region of each subject. We then drew one result (including the original result) from each subject and calculated the group-level mean 1000 times. We calculated the *p* value by counting the number of permutations with equal or higher accuracy than that of the original result and corrected it with Bonferroni correction.

Based on previous studies on tactile softness perception^28^ and matching between sound symbolic words and visual stimuli^16,18^, we selected the following anatomically defined regions as ROIs: the left parietal operculum/insula (PO/Insula, 23,048 mm^3^), right superior frontal gyrus (SFG, limited to y ≥ 0; 82,040 mm^3^), and the region in and around the right posterior superior temporal sulcus (pSTS, limited to y $\leqq$ 0; 39,328 mm^3^). The pSTS only included the middle temporal gyrus and superior temporal sulcus because the superior temporal gyrus can partially overlap the parietal operculum of subjects. These ROIs were anatomically defined using Shattuck’s probabilistic map (LBPA40)^60^ and the probabilistic map in the SPM anatomy toolbox^64–66^.

### Supplementary task 1: word judgment task

We conducted two supplementary tasks to examine the activation patterns when only one type of stimuli (sound symbolic words or touch) was presented: word judgment task and tactile task.

The word judgment task included two within-subject factors: softness and familiarity. The experimental design was identical to that of the matching task except for the following two points; no tactile stimulus was presented, and the subject was asked to estimate the magnitude of softness that is associated with each word. The subject reported the impression of the presented word from 1 (hard) to 4 (soft) by pressing buttons after the stimulus presentation. The order of words was pseudo-randomized such that the same number of words in each category (Familiar_Soft, Familiar_Hard, Unfamiliar_Soft, Unfamiliar_Hard, and Random) were presented in each run. Each word was repeated twice (2 repetitions × 12 words × 5 categories = 120). The design matrix included 6 runs, with each run containing five task-related regressors, each for each category of sound symbolic words.

We conducted whole brain analysis to examine the effect of softness and mean activation of all task conditions against baseline. In the MVPA, a linear SVM was trained on data obtained from 5 runs to predict softness associated with sound symbolic words (softness or hardness) in the remaining run (theoretical chance level = 50%). The remaining procedure was identical to that in the matching task. We analyzed the data of 31 subjects because fMRI data of one subject was not collected due to technical issues.

### Supplementary task 2: tactile task

Finally, the same subjects conducted the tactile task that involved four levels of object compliance. The data was previously reported as “a functional localizer” (Supplemental information in the previous study)^28^. Briefly, the experimental design was identical to that of the matching task, except that no word was presented and the subject was asked to rate the softness of four tactile stimuli. Each stimulus was repeated 4 times in a single run (4 repetitions × 6 runs = 24 trials for each stimulus). The control condition served as an explicit baseline condition where no stimulus was presented. The design matrix included 6 runs, with each run containing two task-related regressors: one for the tactile stimulation, and the other for the baseline condition.

To evaluate the brain activity positively correlated with the subject’s rating of perceived softness (softness-related activation), we performed parametric-modulation analysis^67^ in which the subject’s trial-by-trial ratings were used as parametric modulators. The regressor for parametric modulators was orthogonalized to that for the task-related regressors. In the group analysis, we performed a one-sample t-test on the parameter estimates for parametric modulators that were obtained from the individual analysis. The MVPA procedure was identical to that in the matching task, except that the trained classifier was used to predict softness (4 levels) in the remaining run (theoretical chance level = 25%).

## Supplementary Information

Supplementary Information

## Data Availability

The datasets generated during and/or analyzed during the current study are available from the corresponding author on reasonable request.
