# Supplementary material for: Brain networks underlying the processing of sound symbolism related to softness perception
Source: Sci Rep. 2021 Apr 1;11:7399. doi: 10.1038/s41598-021-86328-6 (PMC8016892; doi:10.1038/s41598-021-86328-6)
Supplement: Supplementary file 1 — Supplementary Information [file 41598_2021_86328_MOESM1_ESM.pdf]

Supplementary Information

Brain networks underlying the processing of sound symbolism related to softness perception

Ryo Kitada, Jinhwan Kwon, Ryuichi Doizaki, Eri Nakagawa, Tsubasa Tanigawa, Hiroyuki Kajimoto, Norihiro Sadato, Maki Sakamoto

***Corresponding author:**

Ryo Kitada, Division of Psychology, School of Social Sciences, Nanyang Technological University, 48 Nanyang Avenue, 639818, Singapore, Tel: +65-6316-8935; E-mail: ryokitada@ntu.edu.sg

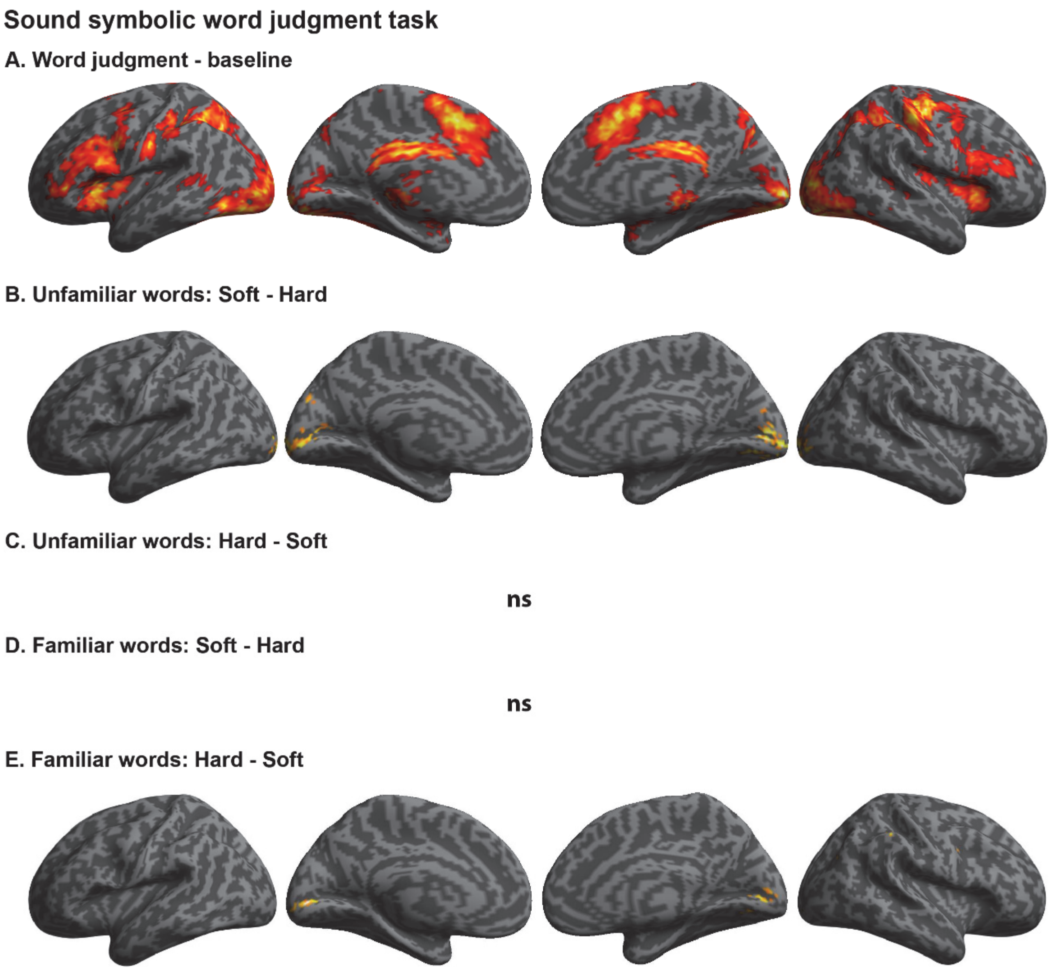

Supplementary Figure 1 Brain activation depicted by the word judgment task

Activation revealed by the contrasts of the conditions in the word judgment task were superimposed on a surface-rendered T1-weighted high-resolution image of an individual unrelated to the study. **(A)** Judgment of softness associated with sound symbolic words was evaluated by comparing mean activation of words judgment against implicit baseline. **(B-E)** Because we found significant interaction between softness and familiarity, we evaluated softness-related activation in each level of familiarity. ns indicates no significant activation. The statistical threshold for the spatial extent test was set at $p < 0.05$, family wise error (FWE) corrected for multiple comparisons over the whole brain when the height (cluster-forming) threshold was set at $p < 0.001$ (uncorrected).

Supplementary Table 1 Sound symbolic words used in the study

"Hard" words		"Soft" words		Random
Unfamiliar	Familiar	Unfamiliar	Familiar	
Gaigai	Bakibaki	Bumyabumya	Fukafuka	Buranebo
Godogodo	Gacchingacchin	Fubafuba	Funwaka	Fugusau
Gokogoko	Gachigachi	Funofuno	Funyafunya	Gakigonu
GoQgoQ	Gachingachin	Fuyufuyu	Fuwafuwa	Iapeso
Gukaguka	Garigari	Munamuna	Fuwari	Jizasaki
Gukoguko	Gichigichi	Munumunu	Hoyahoya	Kosochifu
Gukuguku	Gorigori	Myofumyofu	Kunyakunya	Machijiya
Kadakada	Kachikachi	Myunomyuno	Pafupafu	Nibimuse
Kagukagu	Kachinkachin	Payupayu	Powapowa	Ruwagiku
Kakekake	Kachinkochin	Pohapoha	Poyapoya	Shibaroto
Katokato	Kochikochi	Punopuno	Puyopuyo	Tebahore
Kogukogu	Kochinkochin	Yapuyapu	Yuruyuru	Wakosatu

Note that these words were presented in Japanese Hiragana in the study

Supplementary Table 2 Behavioral performance in the word judgment task

	"Hard" sound symbolic words		"Soft" sound symbolic words		Random
	Unfamiliar	Familiar	Unfamiliar	Familiar	
Rating (SEM)	1.77 (0.04)	1.20 (0.02)	3.42 (0.04)	3.83 (0.03)	2.45 (0.03)
Response time (s) (SEM)	5.86 (0.04)	5.81 (0.04)	5.85 (0.04)	5.81 (0.04)	5.91 (0.04)

SEM, standard error of the mean. Response time is the time relative to the onset of stimulus presentation (time 0 in Figure 1B). Two-way repeated-measures ANOVA (2 levels of softness x 2 levels of familiarity) on response time showed a significant main effect of familiarity [$F(1, 30) = 21.41$, $p < 0.001$]. Neither main effect nor its interaction was significant.

Supplementary Table 3 Brain regions showing matching effects

Spatial extent test		MNI coordinate			Z-values	Location	
Cluster size(mm³)	P-values	x	y	z		Hem	Area
**Matching effects**							
**Congruent vs Incongruent conditions (congruency effect)**							
1352	< 0.001	36	20	0	5.55	R	Insula
1824	< 0.001	2	34	32	5.05	R	Superior frontal gyrus
		6	30	28	4.00	R	Cingulate gyrus
920	< 0.001	-30	24	-2	4.99	L	Insula
424	0.001	-50	42	2	3.31	L	Inferior frontal gyrus[a]
344	0.003	22	-72	-48	4.25	R	Cerebellum
264	0.019	50	12	22	3.77	R	Inferior frontal gyrus[b]
		50	18	20	3.52	R	Inferior frontal gyrus[c]
256	0.023	-38	60	-2	3.82	L	Orbitofrontal cortex
**Incongruent vs Congruent conditions (incongruency effect)**							
n.s.							

The statistical threshold for the spatial extent test was set at P < 0.05, family-wise-error (FWE)-corrected for multiple comparisons over the whole brain when the height (cluster-forming) threshold was set at P < 0.001 (uncorrected). The x, y, and z values represent stereotaxic coordinates (mm). Z values that were transformed from SPM{t} are listed for each set of peak coordinates. Hem, Hemisphere; R, right hemisphere; L, left hemisphere; MNI: Montreal Neurological Institute; n.s., no significant activation. a-d, probability values on cytoarchitectonic maps (Amunts et al., 1999): a 11% for BA45; b 21% for BA44; c 17% for BA45.

Supplementary Table 4 Brain regions showing familiarity effects

Spatial extent test		MNI coordinate				Location	
Cluster size (mm³)	*P*-values	x	y	z	*Z*-value	Hem	Area
**Familiar – Unfamiliar conditions**							
14008	0	-56	-58	18	5.58	L	Angular gyrus
		-58	-58	14	5.08	L	Superior temporal gyrus
		-52	-30	28	4.87	L	Supramarginal gyrus
4920	0	-6	48	18	5.56	L	Superior frontal gyrus
		6	50	8	4.41	R	Superior frontal gyrus
3736	0	-14	-48	36	5	L	Precuneus
		-6	-52	24	4.57	L	Cingulate gyrus
		0	-62	18	3.38	B	Precuneus
2176	0	-50	-16	-10	4.77	L	Superior temporal gyrus
		-54	-24	-12	4.62	L	Middle temporal gyrus
3144	0	64	-46	38	4.75	R	Angular gyrus
		44	-34	20	4.19	R	Supramarginal gyrus
		70	-20	14	3.93	R	Superior temporal gyrus
		66	-16	18	3.87	R	Parietal operculum
472	0.001	40	-6	4	4.73	R	Insula
296	0.015	-34	-34	-16	4.66	L	Fusiform gyrus
		-26	-38	-12	3.45	L	Parahippocampal gyrus
240	0.048	-22	-44	-8	4.57	L	Lingual gyrus
528	0	-70	-42	-6	4.54	L	Middle temporal gyrus
704	0	-38	-2	14	4.51	L	Insula
936	0	-8	-78	-2	4.5	L	Lingual gyrus
1744	0	10	-82	-6	4.46	R	Lingual gyrus
2272	0	10	-90	28	4.39	R	Superior occipital gyrus
		8	-88	24	4.11	R	Cuneus
		12	-94	14	3.41	R	Middle occipital gyrus
248	0.04	-38	-2	-4	4.35	L	Insula
896	0	-6	-18	38	4.29	L	Cingulate gyrus
		-8	-30	46	4.16	L	Precentral gyrus
		-6	-20	46	3.9	L	Superior frontal gyrus
1000	0	-14	-86	34	4.27	L	Superior occipital gyrus
		-4	-80	26	3.91	L	Cuneus
256	0.034	-10	-62	0	4.22	L	Lingual gyrus
368	0.004	-48	-20	22	4.14	L	Postcentral gyrus
		-38	-34	24	3.8	L	Supramarginal gyrus
328	0.008	46	-60	12	3.97	R	Angular gyrus
		58	-62	10	3.17	R	Middle temporal gyrus
304	0.013	38	-10	16	3.93	R	Parietal operculum
		36	2	10	3.89	R	Insula
**Unfamiliar – Familiar conditions**							
528	0	8	24	32	5.43	R	Superior frontal gyrus
7552	0	-36	-90	-6	5.17	L	Middle occipital gyrus
		-32	-92	-12	4.78	L	Inferior occipital gyrus
1416	0	-8	20	44	5.01	L	Superior frontal gyrus

		0	32	40	3.79	B	Superior frontal gyrus
3184	0	-26	-68	22	4.81	L	Superior occipital gyrus
		-28	-44	42	4.39	L	Superior parietal lobule
		-30	-70	24	4.11	L	Middle occipital gyrus
		-30	-64	34	3.5	L	Angular gyrus
1288	0	24	-2	48	4.79	R	Middle frontal gyrus
408	0.002	-26	-64	-28	4.71	L	Cerebellum
584	0	-30	24	0	4.66	L	Insula
4136	0	28	-62	50	4.63	R	Superior parietal lobule
		34	-68	40	4.49	R	Angular gyrus
		32	-66	30	4.21	R	Middle occipital gyrus
		10	-64	48	4.2	R	Precuneus
		28	-70	32	3.9	R	Superior occipital gyrus
3824	0	26	-82	0	4.62	R	Middle occipital gyrus
		34	-84	-8	4.53	R	Inferior occipital gyrus
760	0	-44	12	26	4.37	L	Inferior frontal gyrus
624	0	-46	-40	44	4.14	L	Supramarginal gyrus
464	0.001	36	-64	-28	4.11	R	Cerebellum
296	0.015	-30	0	58	3.92	L	Middle frontal gyrus

The statistical threshold for the spatial extent test was set at P < 0.05, family-wise-error (FWE)-corrected for multiple comparisons over the whole brain when the height (cluster-forming) threshold was set at P < 0.001 (uncorrected). The x, y, and z values represent stereotaxic coordinates (mm). Z values that were transformed from SPM{t} are listed for each set of peak coordinates. Hem, Hemisphere; R, right hemisphere; L, left hemisphere; MNI: Montreal Neurological Institute.

Supplementary Table 5 Brain regions activated by the softness-hardness impression of sound symbolic words

Spatial extent test		MNI coordinate			*Z*-value	Location	
Cluster size (mm³)	*P*-values	x	y	z		Hem	Area
			**Unfamiliar words**				
			**Soft - Hard**				
4536	0	12	-78	-6	5.34	R	Lingual gyrus
		18	-98	-4	5.03	R	Middle occipital gyrus
3488	0	-12	-98	-10	4.67	L	Inferior occipital gyrus
		-8	-96	-4	4.56	L	Lingual gyrus
		-18	-100	-2	4.52	L	Middle occipital gyrus
		-4	-92	4	3.6	L	Cuneus
472	0	6	-78	26	3.67	R	Cuneus
		-2	-80	32	3.65	L	Cuneus
			**Hard - Soft**				
			n.s.				
			**Familiar words**				
			**Soft - Hard**				
			n.s.				
			**Hard - Soft**				
1072	0	0	-94	-4	4.16	B	Lingual gyrus
		-8	-102	-4	3.66	L	Middle occipital gyrus
360	0.002	60	-38	48	4.43	R	Supramarginal gyrus
		50	-42	46	4.05	R	Angular gyrus
976	0	12	-82	4	4.37	R	Lingual gyrus
		6	-92	2	3.16	R	Cuneus
360	0.002	40	4	34	3.86	R	Middle frontal gyrus
		48	8	34	3.77	R	Precentral gyrus
240	0.031	32	-74	32	3.82	R	Middle occipital gyrus

The statistical threshold for the spatial extent test was set at $P < 0.05$, family-wise-error (FWE)-corrected for multiple comparisons over the whole brain when the height threshold was set at $P < 0.001$ (uncorrected). The x, y, and z values represent stereotaxic coordinates (mm). Z values that were transformed from SPM{t} are listed for each set of peak coordinates. Hem, Hemisphere; R, right hemisphere; L, left hemisphere; MNI, Montreal Neurological Institute; n.s., no significant activation.

References

1. Amunts, K. et al. Broca's region revisited: cytoarchitecture and intersubject variability. J Comp Neurol 412, 319-341, doi:10.1002/(sici)1096-9861(19990920)412:2<319::aid-cne10>3.0.co;2-7 (1999).
